# Lower Sensitivity to Happy and Angry Facial Emotions in Young Adults with Psychiatric Problems

**DOI:** 10.3389/fpsyg.2016.01797

**Published:** 2016-11-22

**Authors:** Charlotte Vrijen, Catharina A. Hartman, Gerine M. A. Lodder, Maaike Verhagen, Peter de Jonge, Albertine J. Oldehinkel

**Affiliations:** ^1^Interdisciplinary Center Psychopathology and Emotion regulation (ICPE), University Medical Center Groningen, University of GroningenGroningen, Netherlands; ^2^Interuniversity Center for Social Science Theory and Methodology, Department of Sociology, University of GroningenGroningen, Netherlands; ^3^Behavioural Science Institute, Radboud University NijmegenNijmegen, Netherlands; ^4^Developmental Psychology, University of GroningenGroningen, Netherlands

**Keywords:** facial emotion processing, facial emotion identification, depression, anxiety, avoidant personality problems, attention-deficit hyperactivity disorder, antisocial personality problems, young adults

## Abstract

Many psychiatric problem domains have been associated with emotion-specific biases or general deficiencies in facial emotion identification. However, both within and between psychiatric problem domains, large variability exists in the types of emotion identification problems that were reported. Moreover, since the domain-specificity of the findings was often not addressed, it remains unclear whether patterns found for specific problem domains can be better explained by co-occurrence of other psychiatric problems or by more generic characteristics of psychopathology, for example, problem severity. In this study, we aimed to investigate associations between emotion identification biases and five psychiatric problem domains, and to determine the domain-specificity of these biases. Data were collected as part of the ‘No Fun No Glory’ study and involved 2,577 young adults. The study participants completed a dynamic facial emotion identification task involving happy, sad, angry, and fearful faces, and filled in the Adult Self-Report Questionnaire, of which we used the scales depressive problems, anxiety problems, avoidance problems, Attention-Deficit Hyperactivity Disorder (ADHD) problems and antisocial problems. Our results suggest that participants with antisocial problems were significantly less sensitive to happy facial emotions, participants with ADHD problems were less sensitive to angry emotions, and participants with avoidance problems were less sensitive to both angry and happy emotions. These effects could not be fully explained by co-occurring psychiatric problems. Whereas this seems to indicate domain-specificity, inspection of the overall pattern of effect sizes regardless of statistical significance reveals generic patterns as well, in that for all psychiatric problem domains the effect sizes for happy and angry emotions were larger than the effect sizes for sad and fearful emotions. As happy and angry emotions are strongly associated with approach and avoidance mechanisms in social interaction, these mechanisms may hold the key to understanding the associations between facial emotion identification and a wide range of psychiatric problems.

## Introduction

Facial emotion processing is critical for normal emotional development and engagement in social relationships. Social information gained by processing emotional expressions informs people about the attitudes of others and holds cues for behavioral responses (Salovey and Mayer, 1990; Niedenthal et al., 2000). Therefore, emotion identification is considered to be one of the key elements of successful social interaction. In recent years, many different psychiatric disorders have been associated with emotion-specific biases or general deficiencies in facial emotion identification (Bediou et al., 2012; Kret and Ploeger, 2015). It has been suggested that different problem domains each have their own characteristic condition-specific facial emotion identification biases or deficiencies, which may be useful in early detection and as a target in treatment (Bediou et al., 2012; Isaac, 2012; Penton-Voak et al., 2012, 2013; Rinck, 2013). However, both within and between psychiatric problem domains, large variability exists in the types of emotion identification problems that were reported. The heterogeneous results within specific psychiatric problem domains appear to be due, at least partly, to methodological limitations and differences, for example, small sample sizes, the use of different types of facial emotion processing tasks, and diversity in the study populations regarding combinations of symptoms, symptom severity and comorbidity. This limits the comparability of studies within the same problem domain. Furthermore, most studies only focused on emotion identification deficiencies or biases in one problem domain without excluding participants with co-occurring problems, or adjusting for the presence of these co-occurring problems. This means that the specificity of the facial emotion identification patterns found for a psychiatric problem domain was not addressed. It therefore remains unclear whether patterns found for specific problem domains can be better explained by co-occurrence of other psychiatric problems, or by more general characteristics of psychopathology, for example, problem severity.

Among the implicated problem domains are social anxiety, depression, Attention-Deficit Hyperactivity Disorder (ADHD), and antisocial behavior, with tentative evidence for avoidance behavior. These psychiatric problem domains have been associated with an overall problem with identifying emotions as well as with biased identification of emotions, that is, a heightened or lowered ability to identify specific emotions. For depression, meta-analyses indicate evidence for a bias toward sad faces and away from happy faces (Joormann and Gotlib, 2006; Bourke et al., 2010) and, to a lesser extent, for an overall lower facial emotion identification speed (Bourke et al., 2010; Kohler et al., 2011). In individuals with a history of depression relatively rapid fear identification was found as well (Bhagwagar et al., 2004). Regarding anxiety, a recent meta-analysis showed evidence for a small general emotion identification deficiency in people with social phobia and generalized anxiety (Plana et al., 2014), but it should be noted that emotion-specific effects were ignored, and only total accuracy and intensity scores over all emotions were tested. Notably, other studies reported opposite results, in that people with generalized anxiety tended to perform better at facial emotion identification (Bui et al., 2015). Several studies also found emotion-specific effects: for socially anxious participants a higher sensitivity to angry faces was found (Joormann and Gotlib, 2006), but opposite findings of a lower sensitivity to anger and disgust were also reported (Montagne et al., 2006). ADHD has been associated with overall lower facial emotion identification skills (Sinzig et al., 2008; Rommelse et al., 2011), as well as with more specific problems in identifying sad (Pelc et al., 2006; Aspan et al., 2014; Schönenberg et al., 2015), fearful (Aspan et al., 2014; Schönenberg et al., 2015) and angry emotions (Pelc et al., 2006). Antisocial behavior seems to be primarily related to more difficulties with identifying fear, but has also been associated with difficulties in identifying sadness (Blair et al., 2004; Marsh and Blair, 2008) and subtle happy emotions (Kahler et al., 2012). Avoidance behavior has not been thoroughly investigated, but a first preliminary study suggests that people with avoidant personality problems make more errors in classifying fearful emotions (Rosenthal et al., 2011). Thus, previous findings were heterogeneous, both within and between psychiatric problem domains.

Only few studies have addressed domain-specificity to date. Two studies compared depressed participants, socially anxious participants and healthy controls on their facial emotion identification skills (Gotlib et al., 2004; Joormann and Gotlib, 2006), and found that depressed participants were less capable of identifying subtle happy emotions than the other two groups, whereas participants suffering from social phobia were more proficient in identifying subtle angry emotions than the other two groups. To our best knowledge, no studies explicitly addressed the domain-specificity of emotion identification in a wider range of psychiatric domains. Detailed information regarding the domain-specificity of emotion identification is crucial for achieving a better understanding of the mechanisms underlying different psychiatric problem domains, and may ultimately result in the development of more fine-grained diagnostic tools and treatments.

The aim of the first part of the current study was to investigate whether facial emotion identification bias was related to five different psychiatric problem domains, that is, depressive problems, anxiety problems, avoidance problems, ADHD problems and antisocial problems, in a general population sample of young adults. For all of these problem domains there is evidence of an association with facial emotion identification from previous studies, and the occurrence of these problems in a general population of young adults is also quite common, which is why we considered them the most relevant to investigate and expected sufficient power for all analyses. The advantage of testing all associations in one study is that, due to more methodological homogeneity, the findings for the five domains in our study are more comparable than findings from different studies. The aim of the second part of the study was to determine the domain-specificity of the associations. We did not have hypotheses on domain-specificity in advance because of the lack of previous studies addressing this matter. The two parts of the study are complementary. The first part of the study is aimed at providing more insight into the associations between facial emotion identification and psychiatric problem domains as such, whereas the second part reflects a more mechanistic approach in which unique contributions of single psychiatric problem domains are explored.

We used a so-called ‘morph’ task in which movie clips were shown of neutral faces which gradually changed into full intensity facial emotions (Joormann and Gotlib, 2006; Lodder et al., 2015). The task measured at what intensity participants were able to identify the facial emotion. The use of a morph task enabled us to measure identification of more subtle traces of emotions, which is assumed to give a more ecologically valid perspective than the often used static full intensity facial emotion tasks (Niedenthal et al., 2000; Joormann and Gotlib, 2006). In everyday life full intensity facial emotions are rare but we encounter subtle traces of facial emotions all the time. The benefit of the emotion identification morph task we used is that it enabled us to tap into these frequently occurring everyday life social situations which are essential to social functioning. In addition, because the stimuli gradually change from neutral to full intensity emotions, the morph task was considered a more sensitive instrument than full intensity tasks. High task sensitivity was important in light of our participants; they were not patients with severe psychiatric problems and therefore only subtle alterations in emotion identification were to be expected.

## Materials and Methods

### Sample and Procedure

This study is based on data collected as part of the ‘No Fun No Glory’ project, which investigates anhedonia in young adults. The study was approved by the Medical Ethical Committee from the University Medical Center Groningen (no. 2014/508), participants were treated in accordance with the Declaration of Helsinki and indicated their informed consent online prior to enrolment in the study.

We collected the present data as part of an online survey, for which participants in the northern part of the Netherlands were recruited through advertisements on electronic learning environments of university and higher and intermediate vocational education institutes. We also pitched the study during lectures and classes, and invited participants to participate through flyers and advertisements on social media. After subscribing on the study website^1^, participants received an email with the link to the online survey, containing questionnaires about, for example, pleasure, psychiatric problems and stress. A more detailed description of the questionnaires is available in the ‘No Fun No Glory’ research protocol (van Roekel et al., 2016). Upon finishing the final questionnaire, participants were automatically directed to a facial emotion identification task. After completion of the questionnaire and the task, which, in total, took them on average 35 min, participants received a gift card of 10 Euro and participated in a lottery for fashion vouchers, tablets and a 4-day city trip. Most participants completed the online survey and emotion task at their own preferred time and place, but on a few occasions (<3% of all participants) teachers of intermediate vocational education institutes allowed for the survey and emotion task to be completed in their classroom during regular school hours.

A total of 3,035 participants subscribed to the study website and started the survey. Participants were included in the current study if they had completed both the Adult Self-Report Questionnaire (ASR) and the facial emotion identification task (*N* = 2,620). The task required installing a plugin and attrition between the questionnaire and the task was mostly due to technical problems regarding the plugin. We excluded 43 participants because of suspiciously high error rates or reaction times on the facial emotion identification task, yielding a sample of 2,577 participants. In the description of the task procedures these exclusion criteria are explained in more detail.

The mean age of the participants was 21.4 years (*SD* = 1.9; range 18–27 years) and 78% were females. Most participants attended or had attended university (57%) or higher vocational education (31%), followed by intermediate vocational education (10%) and other types of education (2%).

### Measures and Procedures

#### Psychiatric Problems

The ASR was used to assess psychiatric problems. The ASR is a standardized questionnaire of behavioral and emotional problems, which has been shown to have good reliability and validity (Achenbach and Rescorla, 2003). Responses can be summed to form scale scores on six diagnostic domains based on the Diagnostic and Statistical Manual of Mental Disorders, fourth edition (DSM-IV; American Psychiatric Association, 1994): Depressive problems (14 items), Anxiety problems (seven items), Avoidant personality problems (seven items), Antisocial personality problems (20 items), ADHD problems (13 items) and Somatic problems (nine items). Somatic problems were not included in our study, since there was no theoretical or empirical evidence of the relevance of facial emotion identification for somatic problems. For each problem, answer categories were: 0 = ‘Not True’; 1 = ‘Somewhat or Sometimes True’; 2 = ‘Very True or Often True.’ We divided scale scores by the number of items in the scale so that scores from different problem domains were on the same metric and could be compared easily. In addition to these domain-specific scale scores, a total problems score was calculated for each individual by averaging the mean scores of the five problem domains. In our sample, Cronbach’s alpha’s were 0.83 for the Depressive problems scale, 0.74 for Anxiety problems, 0.79 for Avoidance problems, 0.80 for ADHD problems, 0.66 for Antisocial problems and 0.91 for Total problems.

#### Identification of Facial Emotions

**FIGURE 1:**
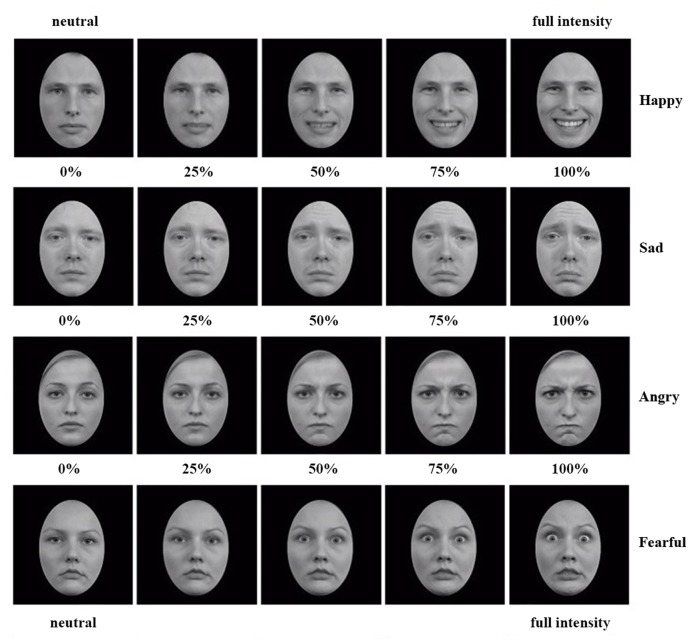
**Examples of the morphs from neutral to full intensity happy, sad, angry, and fearful expressions.** From each movie clip, five of in total hundred frames are presented in this figure.

A morph task developed at Radboud University Nijmegen, the Netherlands (Lodder et al., 2015), was used to assess the emotional intensity of a facial expression required for participants to identify the expressed emotion. In the version we used, stimuli consisted of 24 movie clips that lasted 10 s and contained 100 frames depicting the gradual change (i.e., ‘morph’) from a neutral facial expression to one of four full intensity emotional expressions: happiness, sadness, anger or fear (see **Figure 1** for examples). The movies had a resolution of 256 × 256 pixels, and were created with FaceMorpher (Luxand Inc., Alexandria, VA, USA) from high quality pictures of six different actors (50% females) from the Radboud Faces Database (Langner et al., 2010). Pictures were cropped with an ovoid frame and converted to gray scale to avoid distracting external cues. Four movies were created of each actor, that is, one for each emotional expression. The original task contained 48 movie clips, that is, twelve per facial emotion, whereas we used a shortened version of 24 movie clips, that is, six for each emotion.

The morph task was programmed in Inquisit 4 (Millisecond, Seattle, WA, USA). The task started with the instruction that participants were about to see movies of faces gradually changing from neutral to emotional expressions. Participants were asked to press the space bar as soon as they were able to identify the emotion. After pressing the spacebar the stimulus movie disappeared and participants indicated the emotion they identified by clicking on one of the four emotion labels. After clicking ‘next’ a fixation cross appeared in the middle of the screen for 500 ms, followed by a new stimulus. The order of the movie clips was randomized for each participant separately. Before the start of the actual task participants were shown a complete 10-s example movie, followed by two practice trials. After the practice trials the instructions were repeated, followed by the actual task consisting of 24 trials.

We excluded participants with less than 50% correct answers in total (*N* = 29), because they most likely did not understand the task, were focused on other things, or their strategy consisted of structurally pressing the spacebar before they identified the target emotion. For the remaining participants, the mean reaction time (RT) of correctly identified trials was calculated per emotion. RTs were considered reliable if they were based on at least four movie clips, and hence were calculated only if participants correctly identified at least four out of six movie clips of a specific emotion. This resulted in 6 missing values for RT Happy, 40 for RT Sad, 26 for RT Angry and 52 for RT Fear. Fourteen participants were excluded because they scored the maximum RT (10000 ms) on all four emotions, which indicates that they never pressed the spacebar and just waited until the movie clips stopped. After these spurious RTs were removed, a cut-off for further outliers was defined based on visual inspection of the distribution of the RTs of the four emotions in the complete sample. Because emotion identification differences in the tails of the distribution are likely to be particularly relevant for associations with psychiatric symptoms in a normal population sample, we were rather restrictive and only removed obvious outliers based on visual inspection, that is, RTs that were not connected to the main distribution curve. After the previous steps there were no suspiciously low RTs left upon visual inspection, but we did see indications of high RT outliers and defined mean RT scores higher than or equal to 9800 ms as outliers. The same upper RT limit was used for each emotion to ensure that potential emotion-specific biases would not be removed by removing outliers. Only three participants scored ≥9800 ms on one or more emotions; these high scores were considered as missing values.

### Statistical Analysis

Using SPSS version 22.0, we set out with calculating descriptive statistics of facial emotion identification RTs and ASR DSM problem scores. Next, we performed a series of regression analyses to determine whether the emotional intensity of a facial expression required for its identification, as measured by RTs, was associated with depressive problems, anxiety problems, avoidance problems, ADHD problems, antisocial problems, or total problems. In the first part of the study we performed these analyses for each psychiatric problem domain separately and in the second part we explored the domain-specificity of the associations that were found by adjusting for scores on psychiatric problem domains that had shown similar emotion identification patterns.

#### Part 1: Associations between Facial Emotion Identification RTs and Psychiatric Problems

We regressed ASR DSM problem scores on facial emotion identification RTs. Including facial emotion RTs as independent rather than dependent variables intuitively made more sense to us, but evidently, since our data are cross-sectional, interpretation can never exceed the level of associations and the choice of emotion identification RTs as independent rather than dependent variables is rather arbitrary. We used standardized (*Z*-values) RTs and ASR DSM problem scores to be able to compare regression coefficients across different emotions and problem domains. All analyses consisted of two steps: first, for each problem domain the effects of the RTs for happy, sad, angry, and fearful emotions were tested separately, adjusted for gender and age (i.e., single-emotion models). Because of tentative evidence that identification patterns across multiple emotions may be more relevant to psychiatric problems than identification of individual emotions (Wright et al., 2009; Oldehinkel et al., 2015; Vrijen et al., 2016), in a second step for each problem domain full emotion models were tested including the RTs for all facial emotions (i.e., multi-emotion models), again adjusted for gender and age. Since the dependent variables (i.e., ASR DSM problem scores) and residuals were not normally distributed, we estimated *p*-values from 10,000 bootstrap samples to assure the robustness of our results.

To correct for multiple tests, we used the effective number of tests (Meff; Li and Ji, 2005) as input for the classical False Discovery Rate (FDR) method (Benjamini and Hochberg, 1995). Combining the Meff and the FDR method, as suggested by Li and Ji (2005), enabled us to take into account correlations between tested variables as well as the proportion of significant associations compared to the total number of performed tests. In this way, we corrected for multiple tests without the unwarranted loss of power of more conservative methods. We calculated separate Meffs for the dependent and the independent variables, which were multiplied to obtain the total effective number of tests (i.e., 12; see Supplementary Material for further details). Because we analyzed all emotions separately as well as in full emotion models, and therefore tested all associations twice, we multiplied the effective number of tests by 2 and used a Meff score of 24 as input for the FDR method. The maximum acceptable FDR was set to 0.05, resulting in an FDR-derived significance threshold of 0.0188 for all analyses. Results were only interpreted as significant for *p*-values below this threshold. Further details of this correction for multiple tests as well as all calculations are presented in the Supplementary Material. For FDR calculations, see Supplementary Table S-3.

#### Part 2: Domain-Specificity

Analyses to explore the domain-specificity of associations between facial emotion identification and psychiatric problems were based on patterns found in the results of the analyses as described above. If multiple psychiatric problems domains showed comparable emotion identification patterns (regardless of statistical significance), significant single- and multi-emotion associations from part 1 of the study were re-estimated while adjusting for the problem domains with comparable patterns.

#### Sensitivity Analyses

We performed sensitivity analyses to check whether a different method to define outliers changed the results. There is no consensus on what the best method is for defining RT outliers and recommendations vary between not removing outliers, removing outliers based on absolute cut-offs, removing or trimming outliers based on SDs from the mean, use of transformations, and numerous other methods (Ratcliff, 1993; Erceg-Hurn and Mirosevich, 2008; Whelan, 2008; Wilcox, 2012). The advantage of our restrictive approach to defining outliers is that we most likely only removed spurious RT scores and no genuine RTs. A disadvantage may be that we may not have removed all spurious scores, in which case genuine effects may have remained undetected. Following recommendations of Ratcliff (1993), we applied a second outlier approach as a sensitivity check, that is, we defined outliers as three or more SDs from the mean and trimmed them to the closest non-outlier value; this method was for example used in Lodder et al. (2015). All analyses were repeated and compared to the outcomes which were based on the original outlier selection. We also performed sensitivity analyses to investigate if adjusting for education level changed the results.

## Results

### Descriptive Statistics (**Table 1**)

Mean average scores on all ASR DSM-IV problem domains were between 0 (“not true”) and 1 (“a little true or sometimes true”), as can be expected in a general population study. Females reported on average more depressive, anxiety, avoidance and total problems, whereas males reported slightly more ADHD and antisocial problems. All problem domains were positively correlated, with correlations varying between *r* = 0.28 (avoidance and ADHD problems) and *r* = 0.69 (anxiety and depressive problems). The complete correlation matrix of the psychiatric problem domains is presented in Supplementary Table S-1.

**Table 1:** Descriptive statistics of the main variables in this study.

	Mean (*SD*)		
Variables	Total sample	Males	Females
	*N* = 2522–2577	*N* = 545–566	*N* = 1977–2011
Depressive problems^a^	0.42 (0.33)	0.32 (0.30)	0.45 (0.34)
Anxiety problems^a^	0.58 (0.40)	0.45 (0.36)	0.61 (0.40)
Avoidance problems^a^	0.43 (0.39)	0.39 (0.39)	0.44 (0.39)
ADHD problems^a^	0.44 (0.32)	0.45 (0.32)	0.43 (0.32)
Antisocial problems^a^	0.12 (0.13)	0.15 (0.15)	0.11 (0.12)
Total problems^b^	0.40 (0.24)	0.35 (0.23)	0.41 (0.25)
			


RT happy^c^	4113 (848)	4232 (906)	4080 (828)
RT sad^c^	6542 (1059)	6702 (1093)	6499 (1045)
RT angry^c^	5585 (995)	5816 (1035)	5521 (975)
RT fearful^c^	5861 (1039)	6050 (1107)	5809 (1014)
			


EP happy^d^	1.15 (4.62)	1.48 (5.43)	1.06 (4.37)
EP sad^d^	5.89 (9.59)	7.36 (11.12)	5.49 (9.09)
EP angry^d^	6.06 (9.65)	7.51 (10.44)	5.65 (9.38)
EP fearful^d^	8.37 (11.01)	9.48 (11.20)	8.06 (10.94)

^a^*Mean scores on Adult Self Report (ASR) DSM-IV domains: Depressive problems, Anxiety problems, Avoidant personality problems, Attention Deficit/Hyperactivity (ADHD) problems, Antisocial personality problems. Answer categories: 0 = “not true,” 1 = “a little true or sometimes true,” 2 = “very much true or often true.”*

^b^*Total problems score = average of the sum of mean scores on the above ASR DSM-IV scales.*

^c^*RT = mean reaction time for correct responses measured in milliseconds.*

^d^*EP = mean error proportion.*

**Table 1** contains the mean RTs for identification of the different facial emotions. RTs represent the emotional intensity of the facial expressions required to correctly identify the emotions. Happy emotions were generally identified earlier (i.e., before emotional intensity reached 50%) than the other facial emotions, whereas sad emotions were identified later (i.e., after 65% emotional intensity) than all others. Females required less emotional intensity to identify emotions than males. Correlations among RTs (presented in Supplementary Table S-2) varied from *r* = 0.46 (RT Happy and RT Fearful) to *r* = 0.58 (RT Sad and RT Fearful).

As can be seen from the lower part of **Table 1**, the mean Error Proportions (EPs) for all emotions were low. The mean EP of 7.51 for angry emotions for example indicates that on average participants made identification errors in only 7.5% of the cases, which corresponds to less than half an error out of six possible errors in identifying angry emotions, as an average of one error out of six movie clips would represent a mean EP of 16.67.

### Associations between ASR DSM-IV Problem Domains and Emotion Identification RTs (**Table 2**)

No significant associations were found between depressive or anxiety problems and facial emotion identification RTs. Higher scores on avoidance problems were associated with higher RTs for identifying happiness and anger in the single-emotion models, which means that participants with avoidance problems required relatively intense happy and angry facial emotions in order to identify these emotions. In the multi-emotion model only RT Angry remained significant. Higher scores on ADHD problems were associated with higher RTs for anger in the single-emotion as well as in the multi-emotion model, and higher scores on antisocial problems with higher RTs for happiness in the single-emotion, but not in the multi-emotion model. Finally, higher scores on total problems were associated with higher RTs for happiness and anger in the single-emotion models, whereas only RT Angry remained significant in the multi-emotion model.

**Table 2:** Bootstrapping results of ASR depressive problems, anxiety problems, avoidance problems, ADHD problems, antisocial problems, and total problems regressed on facial emotion identification reaction times.

		RT happy		RT sad		RT angry		RT fearful	
		*B*	*p*-value	*B*	*p*-value	*B*	*p*-value	*B*	*p*-value
Single-emotion models	Depressive problems	0.037	0.061	0.001	0.993	0.040	0.055	0.021	0.312
	Anxiety problems	0.030	0.144	0.001	0.977	0.027	0.188	0.012	0.541
	Avoidance problems	0.055	0.009∗	0.011	0.582	0.061	0.005∗	0.013	0.516
	ADHD problems	0.045	0.035	0.005	0.797	0.056	0.005∗	0.029	0.153
	Antisocial problems	0.057	0.007∗	0.016	0.405	0.037	0.061	0.029	0.145
	Total problems	0.055	0.007∗	0.007	0.725	0.058	0.006∗	0.025	0.241

Multi-emotion models^a^	Depressive problems	0.034	0.153	−0.044	0.102	0.048	0.074	0.004	0.887
	Anxiety problems	0.029	0.225	−0.026	0.343	0.036	0.181	−0.004	0.883
	Avoidance problems	0.048	0.060	−0.045	0.117	0.071	0.012∗	−0.012	0.653
	ADHD problems	0.036	0.150	−0.039	0.149	0.070	0.008∗	−0.006	0.834
	Antisocial problems	0.048	0.045	−0.022	0.380	0.027	0.298	0.002	0.934
	Total problems	0.049	0.045	−0.047	0.087	0.069	0.013∗	−0.006	0.844

Domain-specificity of single-emotion models^b^	Avoidance problems	0.032	0.040			0.038	0.016∗		
	ADHD problems					0.033	0.041		
	Antisocial problems	0.030	0.090						

Domain-specificity of multi-emotion models^c^	Avoidance problems					0.044	0.035		
	ADHD problems					0.047	0.025		

*ASR, Adult Self-report; ADHD, Attention/Deficit Hyperactivity Disorder; RT, mean reaction time for correct responses in milliseconds; all variables were standardized (*Z*-values), therefore *B*s can be interpreted as *β*s; all effects were adjusted for gender and age; all *p*-values were estimated from 10,000 bootstrap samples.*

∗ *p-value < multiple test correction significance threshold of 0.0188.*

^a^*Multi-emotion models included RTs for all four emotions.*

^b^*Significant single-emotion associations (*p* < 0.0188) adjusted for all other specific psychiatric problem domain scores.*

^c^*Significant multi-emotion associations (*p* < 0.0188) adjusted for all other specific psychiatric problem domain scores.*

### Domain-Specificity

Statistically significant results were found for the specific psychiatric domains of avoidance, ADHD and antisocial problems (see upper part of **Table 2**). To explore the domain-specificity of these findings we repeated the analyses of the significant associations as reported in the upper part of **Table 2**, this time adjusting for the scores on all problem domains that showed similar emotion identification patterns. Regardless of statistical significance, a general emotion identification pattern seemed to be present for all problem domains, that is, the highest absolute *B*s belonged to RT Happy and RT Angry in all single-emotion models for all domains, and all of these *B*s were positive, suggesting patterns in similar directions. In other words, all psychiatric problem domains showed the same pattern in that they were all more strongly associated with RTs for happy and angry emotions than with RTs for sad and fearful emotions. Therefore, to explore the domain-specificity of the reported significant associations, the single-emotion analyses for avoidance, ADHD and antisocial problems and the multi-emotion analyses for avoidance and ADHD problems were repeated while adjusting for all other specific problem domains.

A comparison of the regression coefficients (*B*s) of the single- and multi-emotion models (upper part of **Table 2**) to the estimates of the domain-specificity analyses (lower part of **Table 2**) showed similar patterns for all three problem domains. Between 53 and 67% of each regression coefficient remained after adjusting for the other problem domains.

### Sensitivity Analyses

#### Outlier Selection Method

The use of an alternative method to handle outliers, with outliers defined as deviating three or more SDs from the mean and trimmed to the closest non-outlier value, did not change the results of the analyses: estimates and *p*-values of were comparable to **Table 2**, with only small differences. The same results as before remained significant below the multiple test correction threshold (data available upon request).

#### Education Level

Adjusting for education level, *B*s generally decreased. Fewer *p*-values reached statistical significance at α = 0.05 compared to the *p*-values in our main analyses, and a new multiple test correction significance threshold which was calculated based on the results adjusted for education level left none of the effects statistically significant. Regardless of statistical significance, the general patterns of the associations between facial emotion identification and psychiatric problems were similar to those without adjusting for education level. For exact regression coefficients and *p*-values, see Supplementary Table S-4.

## Discussion

The aim of this study was to examine associations between facial emotion identification and psychiatric problems in young adults, and to explore the domain-specificity of these associations. We used a morph task in which movie clips were presented that gradually morphed from a neutral facial expression to a full intensity facial emotion.

Following expectations, we found associations between facial emotion identification and psychiatric problems. More specifically, young adults with avoidance problems required more intense happy and angry facial emotions to correctly identify these emotions, that is, they were less sensitive to subtle happy and angry emotions. Furthermore, antisocial problems were mainly associated with lower sensitivity to happy facial emotions, and ADHD with lower sensitivity to angry emotions. Contrary to what we expected, we did not find associations between facial emotion identification and depressive or anxiety problems. We found emotion-specific biases, but there was no evidence for overall emotion identification deficiencies. The effects we found could not be fully explained by co-occurring psychiatric problems. Whereas this seems to indicate domain-specificity, inspection of the overall pattern of effect sizes regardless of statistical significance revealed generic patterns as well, in that for all psychiatric problem domains the effect sizes for happy and angry emotions were larger than the effect sizes for sad and fearful emotions. For each problem domain the findings will be discussed in more detail below, followed by a discussion of the domain-specificity, sensitivity analyses, strengths and limitations, and suggestions for future research.

### Facial Emotion Identification in Different Psychiatric Problem Domains

#### Avoidance Problems

Our finding that experiencing avoidance problems was associated with lower sensitivity to happy and angry emotions may be explained by approach-avoidance mechanisms. It has been argued that happy facial emotions are naturally rewarding social stimuli which elicit approach behavior and that there is a bias toward happy faces in the general population (Evans et al., 2011; Furl et al., 2012). Angry facial emotions are commonly regarded as threatening and they evoke avoidance (Seidel et al., 2010). Previous findings further suggest that the avoidance system elicits withdrawal behavior and inhibits goal-directed behavior (Corr, 2001; Leventhal, 2008), and that people suffering from avoidance problems tend to rate other people as more rejecting and less friendly (Meyer et al., 2004). More avoidance behavior in response to angry faces and less approach behavior in response to happy faces may have resulted in a lower sensitivity to happy and angry emotions during the emotion identification task. Subtle traces of anger may not be picked up if anger is more strongly avoided and subtle traces of happiness may not be picked up if the urge to approach happiness is weaker.

In the only other study that explicitly addressed the relation between avoidance problems and facial emotion identification, adults meeting full diagnostic criteria of DSM-IV Avoidant Personality Disorder (APD) made more errors in classifying full intensity fearful emotions during a morph task, but did not differ from controls in terms of sensitivity (Rosenthal et al., 2011). This is in contrast with our finding that avoidance problems are associated with lower sensitivity to facial happiness. There is less discrepancy with our finding that avoidance problems are associated with lower sensitivity to anger, since fear and anger are both negative emotions that have both been found to evoke avoidance in socially anxious individuals (Stirling et al., 2006; Rosenthal et al., 2011). Several methodological differences between our study and the one by Rosenthal et al. (2011) could be responsible for the contrasting results on sensitivity to happiness: whereas in our study participants were only asked to differentiate between the four basic emotions, the task used by Rosenthal et al. (2011) also contained disgust and surprise as facial emotions. Importantly, their sample consisted of only 17 adults with APD and 16 controls. The general pattern of their reported average sensitivity scores per emotion per group suggests that the use of a larger sample may in fact have resulted in significant associations for sensitivity to happiness and anger in the same direction as found in our study.

#### Antisocial Problems

Antisocial problems have frequently been associated with poor identification of sad or fearful facial emotions (Blair et al., 2004; Marsh and Blair, 2008). Lack of empathy is often used as an explanation for poor identification of sad emotions and an ability to experience fear as an explanation for poor identification of fearful emotions. In our study we found no evidence of associations between antisocial problems and identification of sad or fearful facial emotions. We did find that participants with antisocial problems were less sensitive to happy emotions, which has been occasionally but not consistently found in other studies (Kahler et al., 2012). Lack of empathy or an inability to experience fear cannot explain this finding, however, a third characteristic of antisocial problems may. That is, antisociality is also characterized by a lower appreciation of social interactions. As was suggested by Kahler et al. (2012), being unable to identify more subtle traces of happy emotions may contribute to experiencing social interactions as unsupportive and stressful, which may reinforce hostile attitudes. Reversely, low appreciation of social interactions may lead to inattention to rewarding social cues such as smiling faces, thereby blocking the possibility of positive reinforcement. It is more difficult to explain why we did *not* find associations between antisocial problems and identification of sad and fearful emotions. As Blair et al. (2004) and Marsh and Blair (2008) studied people with more severe antisocial problems than we did in the current study, we could speculate that perhaps lack of empathy and an inability to experience fear are symptoms associated with more severe antisocial problems, which would explain why we did not find associations between antisocial problems and identification of sad and fearful emotions.

#### ADHD Problems

In previous studies broad facial emotion processing deficiencies in relation to ADHD have been reported (Sinzig et al., 2008; Rommelse et al., 2011) as well as emotion-specific biases (Pelc et al., 2006; Aspan et al., 2014; Schönenberg et al., 2015). In the current study, which differed from the previous ones in that we investigated young adults instead of children, and assessed ADHD problems in a non-referred sample by means of self-reports rather than in patients with formal diagnoses, we found an association with angry but not the other three emotions. Less proficiency in identifying angry facial emotions has been reported before (Singh et al., 1998; Pelc et al., 2006), but not consistently. A possible explanation could be that children with ADHD have learned to avoid anger when it is expressed to them, or miss important cues necessary for identifying angry expressions altogether (Singh et al., 1998; Pelc et al., 2006). The latter explanation is in line with results from studies using event-related potentials and skin conductance responses, which suggest that children with ADHD show less sensitivity to punishment (Masunami et al., 2009; van Meel et al., 2011). Although speculative, findings may also link to in relation to ADHD reported neural activation (Cubillo et al., 2012) and connectivity (Tomasi and Volkow, 2012) differences in lateral orbitofrontal cortex, an area which has been implicated in the evaluation of punishment (O’Doherty et al., 2001; Kringelbach and Rolls, 2004). However, there is no convincing body of evidence for these findings. Because we only found evidence that ADHD is associated with problems in identifying anger, it is unlikely that our findings are due to a general problem with concentration on the task for participants with heightened ADHD problems, as this would have resulted in identification problems on all emotions.

#### Depressive and Anxiety Problems

Contrary to expectations, no associations were found for depressive and anxiety problems. Although the results were not significant, participants who reported depressive and anxiety problems tended to show patterns similar to those of avoidance problems, that is, experiencing more problems was associated with being less sensitive to happy and angry emotions. Since avoidance behavior plays an important role in depressive and anxiety disorders (Ottenbreit and Dobson, 2004; Chawla and Ostafin, 2007; Ottenbreit et al., 2014), the associations between depressive or anxious problems and emotion identification found in previous studies, which were often conducted in clinical patient populations, may have been at least partly driven by avoidance problems. Though speculative, this explanation would be consistent with our lack of findings for depressive and anxiety problems, since the ASR depressive and anxiety scales contain hardly any avoidance items whereas in clinical patients avoidance problems are part and parcel of depression and anxiety disorders.

### Domain- and Emotion-Specificity

Our results indicate that co-occurring problems in other psychiatric domains can only partly explain the effects found for avoidance problems, ADHD problems and antisocial problems. Whereas this seems to indicate domain-specificity, for comparing emotion identification patterns between the different domains it makes more sense to compare effect sizes than *p*-values. Inspection of the overall pattern of effect sizes regardless of statistical significance suggests that all psychiatric problem domains were more strongly associated with sensitivity to happy and angry emotions than with sensitivity to sad and fearful emotions. A possible explanation would be that the variation in emotion identification skills within each of the psychiatric problem domains is caused by different mechanisms, and that these mechanisms coincidentally resulted in similar emotion identification patterns. Another explanation would be the presence of a single mechanism that underlies the variation in facial emotion identification across all psychiatric problem domains. We propose that our findings may reflect a generic association of psychiatric problems with less approach tendencies in response to happy facial emotions and more avoidance tendencies in response to angry facial emotions. Variation in the role approach and avoidance problems play in the different problem domains could explain domain-specific variations in effect size as well as why effects for either happy or angry emotions are more pronounced in certain domains.

A closer look at the function of happy and angry facial emotions, and how they are different from sad and fearful emotions, may help to explain our finding that only identification of happy and angry emotions was associated with psychiatric problems. Although happy and angry facial emotions elicit opposite responses, that is, approach and avoidance (Marsh et al., 2005; Seidel et al., 2010; Evans et al., 2011; Furl et al., 2012), there are also commonalities. Happiness and anger are both strong and relatively unambiguous facial emotions associated with direct relevance to the perceiver. Sadness and fear are often less directly aimed at an individual and are more ambiguous in their relevance to the perceiver. These differences are supported by studies showing that happy and angry emotions are identified faster than sad or fearful ones (Williams et al., 2005; Calvo and Marrero, 2009) and that, in frontal view, directly facing participants, happy and angry emotions are identified more easily, whereas sad and fearful emotions are better identified when gazing in a different direction (Adams and Kleck, 2005). Happy and angry emotions presented in frontal view are also found to elicit stronger emotional responses than when presented in averted gaze direction (Sato et al., 2010). This would imply that, from the perspective of our participants, happy and angry expressions differ from sad and fearful ones in that they are most strongly and unambiguously associated with approach and avoidance tendencies in relation to the person expressing these emotions. If, as we proposed, approach and avoidance mechanisms are indeed underlying the associations between facial emotion identification and psychiatric problems, it seems plausible that differences are found particularly regarding happy and angry expressions, since these most unambiguously evoke strong approach and avoidance tendencies. Please note that we only offered frontal view stimuli, which potentially increased the salience of happy and angry emotions, but cannot test whether averted gaze directions would have changed the results of this study.

### Education Level

Education level explained part of the variation in psychiatric problems, that is, individuals with low levels of education reported more psychiatric problems, and the already small main effects of facial emotion identification on psychiatric problems weakened. Education level is a rough proxy for intelligence, parental socioeconomic status and the social environment combined. Therefore, we can only speculate on why education level and facial emotion identification explained partly the same variation in psychiatric problems. It is possible that education level or intelligence is partly confounding the relationship between facial emotion identification and psychiatric problems, for example if low intelligence or low education level renders an individual vulnerable to psychiatric problems and also explains slower responses to a facial emotion identification task. An alternative explanation is that the presence of psychiatric problems influences educational attainment, as was found in several studies (McLeod and Fettes, 2007; Veldman et al., 2014), as well as facial emotion identification.

### Strengths and Limitations

The large sample size of our study enabled us to consider multiple problem domains at once, using similar methods and instruments for all domains, and explore the domain-specificity and emotion-specificity of associations between facial emotion identification and psychiatric problems. A methodological strength of our study is that it gives an indication of the validity of online assessment of the morph task. As far as we know we are the first to use an online facial emotion identification morph task that is completed in participants’ own environment without a researcher present. The emotion identification patterns and mean reaction times we found are highly similar to the ones reported in a study among 173 female undergraduate students, in which a 48-video-clip version of the morph task was assessed in a laboratory situation (Lodder et al., 2015). In our study the variation was slightly higher for all emotions, with the largest difference for happy emotions. The comparability of our results suggests that the shortened 24-video-clip version of the facial emotion identification task produces valid results, also when assessed online outside the lab without an instructor present. This is promising for future research since it reduces practical constraints for assessing larger groups of people on multiple occasions.

Our study also has several limitations, warranting that the findings should be interpreted with caution. First, our results apply to a general population of young adults and results cannot be generalized to more severely affected clinical populations. However, the variation in psychiatric problems in our sample suggests that the small effects we found are not due to accidental recruitment of an overly healthy group. Fifteen percent of the participants experienced psychiatric problems at a clinical level on at least one of the five psychiatric problem domains, 12% experienced problems at a subclinical level and 73% remained within the normal range for all problem domains (Achenbach and Rescorla, 2003). For the separate problem domains, between 1% (antisocial problems) and 8% (avoidance problems) of the participants experienced clinical levels of psychiatric problems and between 1% (antisocial problems) and 9% (depressive problems) experienced psychiatric problems at subclinical levels. We therefore propose that only small effects can be expected in a general population of young adults, but whether this also holds for clinical populations remains to be determined. A second limitation is that psychiatric problems were assessed by means of the ASR, and particularly for ADHD problems self-report is not the gold standard. More valid ADHD scores would have been established if parent and teacher reports had been taken into account as well, but these were not collected.

### Future Research

First of all, our approach of comparing identification of multiple facial emotions in multiple psychiatric problem domains should be repeated in clinical samples that are sufficiently large to allow adjusting for co-occurring psychiatric symptoms. In more severely affected patient groups, more pronounced emotion identification patterns may be found with clearer implications of underlying mechanisms. A large advantage of investigating multiple problem domains in one study *per se* is that due to more methodological homogeneity, the findings for different domains are better comparable than findings between different studies. Second, more research is needed to identify the underlying mechanisms of associations between facial emotion identification and psychiatric problems. A potentially viable direction for future research would be a multidisciplinary investigation of the role of approach and avoidance mechanisms in relation to emotion identification in different psychiatric domains, perhaps starting with clinical depression and anxiety, since these have been most consistently associated with approach and avoidance problems (Ottenbreit and Dobson, 2004; Chawla and Ostafin, 2007; Ottenbreit et al., 2014). Combining behavioral measures with neurobiological correlates of approach and avoidance tendencies in response to facial emotions in the different psychiatric domains seems particularly relevant for understanding the underlying mechanisms. The amygdala and associated circuitry have been primarily related to avoidance mechanisms, and the striatum and associated circuitry to approach mechanisms (Ernst and Fudge, 2009). Furthermore, amygdala and striatum abnormalities have been associated with different psychiatric problem domains, for example with depression (Stuhrmann et al., 2011), (social) anxiety (Shin and Liberzon, 2009; Freitas-Ferrari et al., 2010), avoidance behavior (Schlund et al., 2013), ADHD (Cubillo et al., 2012; Tajima-Pozo et al., 2016) and antisocial behavior (Glenn and Yang, 2012; Blair, 2013). To further link approach and reward mechanisms to facial emotion processing in the specific psychiatric problem domains, we commend future study of activation and connectivity of the amygdala, striatum and their associated circuits during the processing of conscious as well as subliminally presented facial emotions. It has already been found in several studies that depressed patients showed greater amygdala responses for sad than for happy subliminally presented faces whereas healthy controls showed greater responses to the happy faces (Suslow et al., 2010; Victor et al., 2010; Stuhrmann et al., 2013). Third, consideration of the different types of facial emotion identification errors, for example, mistaking happy faces for sad faces or sad faces for angry faces, may also provide new insights. The morph task, which focuses on the time it takes to identify an emotion, is not equipped to investigate the different types of errors; since errors are rare there is insufficient power to consider the different error types. Studies that use other paradigms, wherein participants are offered pictures or movie clips of specific morphing stages using a forced choice paradigm, are better suitable for this type of research.

More in general, there is a large diversity of findings related to facial emotion identification and psychiatric problems. New studies, as the current one, only seem to increase this heterogeneity, and the small effect sizes reported in our study are a rule rather than exception, especially in general population studies. Although effects reported in clinical populations are generally slightly larger, the diversity of results equally applies and clinical relevance is difficult to establish. Continuing to investigate associations between DSM-based psychiatric domains and facial emotion identification may not be the way to proceed toward clinically relevant findings in the future. In the perspective of recent calls for a revision of the categorical DSM system into a more dimensional approach to psychiatry (Kendler, 2012), it may be more beneficial to focus on smaller units of psychopathology, for example symptoms or clusters of symptoms. In support of this, we previously found that facial emotion identification was a stronger predictor of symptoms of anhedonia than of depression itself (Vrijen et al., 2016). Additionally, a potentially viable way to proceed would be to use facial emotion identification biases or deficiencies rather than psychiatric diagnoses or symptoms as the starting point, and from this perspective investigate associations between *extreme* emotion identification biases or deficiencies and psychiatric symptoms. It seems quite plausible that individual differences in facial emotion identification are not associated with psychiatric problems as long as they remain within a certain range. Comparing a group with extreme deviations in emotion identification to a ‘normal range’ group may produce more clinically relevant findings and may potentially indicate new mechanisms underlying associations between facial emotion identification and domain-transcendent combinations of psychiatric symptoms.

## Author Contributions

CV, conception and design of the study, data collection, data analysis and interpretation, drafted, and revised the manuscript; AO, CH, and PdJ, conception and design of the study, data interpretation, drafted, and revised the manuscript. GL and MV, development of the facial emotion identification task and revised the manuscript. All authors have made substantial contributions to the manuscript and approved it for publication.

## Conflict of Interest Statement

The authors declare that the research was conducted in the absence of any commercial or financial relationships that could be construed as a potential conflict of interest.

## Footnotes

1 www.nofunnoglory.nl
