# Supplementary material for: Lower Sensitivity to Happy and Angry Facial Emotions in Young Adults with Psychiatric Problems
Source: Front Psychol. 2016 Nov 22;7:1797. doi: 10.3389/fpsyg.2016.01797 (PMC5118561; doi:10.3389/fpsyg.2016.01797)
Supplement: Supplementary file 1 [file Data_Sheet_1.pdf]

Supplementary Material

Lower sensitivity to happy and angry facial emotions in young adults with psychiatric problems

C. Vrijen*, C.A. Hartman, G.M.A. Lodder, M. Verhagen, P. de Jonge, A.J. Oldehinkel

*** Corresponding Author**: c.vrijen@umcg.nl

1 Multiple test correction method: False Discovery Rate combined with Effective number of tests

To correct for multiple tests, the effective number of tests (Meff) (Li and Ji, 2005) was calculated and used as input for the classical False Discovery Rate (FDR) method (Benjamini and Hochberg, 1995). The Meff and the FDR method were both developed as instruments to correct for multiple testing while remaining sufficient power, but they are based on different principles. The main idea behind the Meff method is that the effective number of tests can be determined by means of the correlations between tested variables. If correlations are higher, the number of effective tests decreases and correcting for the total rather than the effective number of tests is too conservative and results in unwarranted loss of power. Characteristic of the FDR method is that power decreases when the number of significant results decreases. The main thought behind this approach is that finding one significant result in 20 tests calls for a more stringent correction than finding 10 significant results in 20 tests. The advantage in power of FDR above more conservative methods increases when more significant results are found and when the total number of tests increases. Combining the Meff and the FDR method, as suggested by a.o. Li and Ji (2005), seemed appropriate for our study since we expected high correlations between our dependent variables, moderate correlations between our independent variables, and based on previous findings we also expected to find multiple significant results.

1.1 Meff backgrounds and calculation method

The Li and Ji Meff is an adaptation of the Cheverud method (Cheverud, 2001). Li and Ji claim that Cheverud's Meff is still too conservative and is only appropriate for two extreme cases, i.e. cases of high correlations between tested variables and cases with hardly any correlations between tested variables, and not for studies with many tests and moderate correlations between tested variables. According to Li and Ji their method gives a more accurate estimate of the Meff that works in the extreme cases as well as in the continuum between these extremes. Especially in the continuum Cheverud's Meff is claimed to be overly large and in this area the Li and Ji Meff would result in more power.

Although the Meff is often calculated only for dependent variables, since we aimed at correcting for the number of independent variables as well, we calculated separate Meffs for the dependent and the independent variables and then multiplied them to establish the total number of effective tests. First, the correlation matrix of our six dependent variables was used to calculate eigenvalues for each of these variables, by using the application offered on www.junningli.org. Subsequently, the following equation, as proposed by Li and Ji (2005), was applied to the eigenvalues:

$$\begin{cases} \text{Meff} = \sum_{i=1}^{M} f(|\lambda i|) \\ f(x) = I(x \geq 1) + (x - \lfloor x \rfloor), x \geq 0 \end{cases}$$

Eigenvalues are decomposed into an **integral part**, with $I(x \geq 1)$ representing what should be counted as 1 test, and a *nonintegral* part $x - \lfloor x \rfloor$, representing what counts as a partial test.

The Meff was first calculated for the dependent variables. This resulted in a $\text{Meff}_{\text{dependent}}$ score of 4 (see Table S-1).

Table S-1

Correlation matrix dependent variables, eigenvalues and Meff$_{dependent}$

	Correlations						Eigenvalues	Meff
	Depressive	Anxiety	Avoidance	ADHD	Antisocial	Total prob.		
Depressive	1.000	0.694	0.635	0.521	0.407	0.882	**3**.*8675*	1.8675
Anxiety	0.694	1.000	0.573	0.405	0.311	0.837	**0**.*9350*	0.9350
Avoidance	0.635	0.573	1.000	0.281	0.284	0.784	**0**.*5357*	0.5357
ADHD	0.521	0.405	0.281	1.000	0.495	0.689	**0**.*3959*	0.3959
Antisocial	0.407	0.311	0.284	0.495	1.000	0.539	**0**.*2694*	0.2694
Total prob.	0.882	0.837	0.784	0.680	0.539	1.000	**0**.*0000*	0.0000 +
Meff$_{dependent}$								**4.0035**

The same procedure was followed for the four independent variables, resulting in a Meff$_{independent}$ score of 3 (see Table S-2).

Table S-2

Correlation matrix independent variables, eigenvalues and Meff$_{independent}$

	Correlations				Eigenvalues	Meff
	RT Happy	RT Sad	RT Angry	RT Fear		
RT Happy	1.000	0.469	0.480	0.459	**2**.*5384*	1.5384
RT Sad	0.469	1.000	0.557	0.576	**0**.*5738*	0.5738
RT Angry	0.480	0.557	1.000	0.530	**0**.*4691*	0.4691
RT Fear	0.459	0.576	0.530	1.000	**0**.*4187*	0.4187+
Meff$_{independent}$						**3.000**

Multiplying Meff$_{dependent}$ and Meff$_{independent}$ resulted in a total of 12 effective tests. Because for all problem domains we analyzed all emotions separately as well as in full emotion models and therefore tested all of them twice, we multiplied the effective number of tests by 2 and used a Meff score of 24 as input for the FDR method.

1.2 FDR backgrounds and calculations (Table S-3)

For the calculation of the classical FDR (Benjamini and Hochberg, 1995), first the p-values of all performed statistical tests are ranked from low to high. Subsequently, with alpha set to 0.05, for each found result an FDR corrected significance threshold is calculated:

$$FDR\ derived\ significance\ treshold = \frac{0.05}{number\ of\ tests / ranking}$$

When these are calculated, it is determined which of the original p-value is still smaller than the FDR corrected significance threshold. Each result with that ranking or higher is still considered significant after multiple testing. We combined FDR and Meff and therefore replaced the number of tests by the Meff-value:

$$FDR\ derived\ significance\ treshold = \frac{0.05}{Meff / ranking}$$

As can be seen in the table below, for the hypothesis with the 9th p-value ranking the p-value is still below the FDR-derived significance threshold, but this is no longer the case for the 10th p-value. The FDR-derived significance thresholds for rank 9 can be calculated as follows:

$$\frac{0.05}{24/9} = 0.01875$$

Since the 9th p-value is the last one to remain below the FDR-derived significance threshold, this significance threshold (0.01875) is the threshold for all tests.

Table S-3

Effective number of tests (Meff = 24) applied to FDR classical method, with alpha set to 0.05

**Hypothesis name**	***p*-value**	**Rank**	**Ascending p-values**	**Hypothesis name**	**FDR-derived significance thresholds**	**FDR-adjusted *p*-values**
Happy depres	0.061	1	0.005	Angry adhd*	0.002083	0.030857
Sad depres	0.993	2	0.005	Angry avoi*	0.004167	0.030857
Angry depres	0.055	3	0.006	Angry total*	0.006250	0.030857
Fear depres	0.312	4	0.007	Happy total*	0.008333	0.030857
Happy anx	0.144	5	0.007	Happy antisoc*	0.010417	0.030857
Sad anx	0.977	6	0.008	Angry adhd multi*	0.012500	0.030857
Angry anx	0.188	7	0.009	Happy avoi*	0.014583	0.030857
Fear anx	0.541	8	0.012	Angry avoi multi*	0.016667	0.034667
Happy avoi	0.009	9	0.013	Angry total multi*	0.018750	0.034667
Sad avoi	0.582	10	0.035	Happy adhd	0.020833	0.084000
Angry avoi	0.005	11	0.045	Happy total multi	0.022917	0.090000
Fear avoi	0.516	12	0.045	Happy antisoc multi	0.025000	0.090000
Happy adhd	0.035	13	0.055	Angry depres	0.027083	0.091500
Sad adhd	0.797	14	0.060	Happy avoi multi	0.029167	0.091500
Angry adhd	0.005	15	0.061	Angry antisoc	0.031250	0.091500
Fear adhd	0.153	16	0.061	Happy depress	0.033333	0.091500
Happy antisoc	0.007	17	0.074	Angry depres multi	0.035417	0.104471
Sad antisoc	0.405	18	0.087	Sad total multi	0.037500	0.116000
Angry antisoc	0.061	19	0.102	Sad depres multi	0.039583	0.128842
Fear antisoc	0.145	20	0.117	Sad avoi multi	0.041667	0.140400
Happy total	0.007	21	0.144	Happy anx	0.043750	0.141231
Sad total	0.725	22	0.145	Fear antisoc	0.045833	0.141231
Angry total	0.006	23	0.149	Sad adhd multi	0.047917	0.141231
Fear total	0.241	24	0.150	Happy adhd multi	0.050000	0.141231
Happy depres multi	0.153	25	0.153	Happy depres multi	0.052083	0.141231
Sad depres multi	0.102	26	0.153	Fear adhd	0.054167	0.141231
Angry depres multi	0.074	27	0.181	Angry anx multi	0.056250	0.160889
Fear depres multi	0.887	28	0.188	Angry anx	0.058333	0.161143
Happy anx multi	0.225	29	0.225	Happy anx multi	0.060417	0.186207
Sad anx multi	0.343	30	0.241	Fear total	0.062500	0.192800
Angry anx multi	0.181	31	0.298	Angry antisoc multi	0.064583	0.230710
Fear anx multi	0.883	32	0.312	Fear depress	0.066667	0.234000
Happy avoi multi	0.060	33	0.343	Sad anx multi	0.068750	0.249455
Sad avoi multi	0.117	34	0.380	Sad antisoc multi	0.070833	0.268235
Angry avoi multi	0.012	35	0.405	Sad antisoc	0.072917	0.277714
Fear avoi multi	0.653	36	0.516	Fear avoi	0.075000	0.344000
Happy adhd multi	0.150	37	0.541	Fear anx	0.077083	0.350919
Sad adhd multi	0.149	38	0.582	Sad avoi	0.079167	0.367579
Angry adhd multi	0.008	39	0.653	Fear avoi multi	0.081250	0.401846
Fear adhd multi	0.834	40	0.725	Sad total	0.083333	0.435000
Happy antisoc multi	0.045	41	0.797	Sad adhd	0.085417	0.466537
Sad antisoc multi	0.380	42	0.834	Fear adhd multi	0.087500	0.471070
Angry antisoc multi	0.298	43	0.844	Fear total multi	0.089583	0.471070
Fear antisoc multi	0.934	44	0.883	Fear anx multi	0.091667	0.473067
Happy total multi	0.045	45	0.887	Fear depres multi	0.093750	0.473067
Sad total multi	0.087	46	0.934	Fear antisoc multi	0.095833	0.487304
Angry total multi	0.013	47	0.977	Sad anx	0.097917	0.496500
Fear total multi	0.844	48	0.993	Sad depress	0.100000	0.496500

* Significant after multiple test correction

2 Post hoc sensitivity analyses: adjusting for education level

Table S-4

Bootstrapping results of ASR Depressive Problems, Anxiety problems, Avoidance problems, ADHD problems, Antisocial problems and Total problems regressed on facial emotion identification reaction times, ***adjusted for education level***

		RT Happy		RT Sad		RT Angry		RT Fearful	
		B	*p*-value	*B*	*p*-value	*B*	*p*-value	*B*	*p*-value
**Single emotion models**	Depressive problems	.029	.136	-.006	.749	.032	.119	.010	.634
	Anxiety problems	.026	.200	-.003	.895	.024	.250	.007	.727
	Avoidance problems	.051	.015	.007	.728	.056	.008	.007	.753
	ADHD problems	.033	.118	-.005	.809	.044	.028	.011	.578
	Antisocial problems	.042	.043	.003	.860	.020	.298	.006	.774
	Total problems	.046	.026	-.001	.953	.048	.020	.011	.607
**Multi emotion models**[a]	Depressive problems	.031	.203	-.041	.123	.045	.093	-.005	.845
	Anxiety problems	.028	.254	-.024	.368	.034	.195	-.008	.756
	Avoidance problems	.046	.071	-.043	.133	.069	.015	-.018	.506
	ADHD problems	.031	.220	-.035	.190	.065	.012	-.020	.437
	Antisocial problems	.041	.090	-.017	.505	.021	.419	-.017	.520
	Total problems	.045	.067	-.044	.112	.065	.018	-.017	.533

ASR = Adult Self-report, ADHD = Attention/Deficit Hyperactivity Disorder; RT = mean reaction time for correct responses in milliseconds; all variables were standardized (Z-values), therefore *B*s can be interpreted as *β*s; all effects were adjusted for gender, age and education level; all *p*-values were estimated from 10,000 bootstrap samples

No *p*-values < multiple test correction significance thresholds

[a] Multi emotion models contained all four emotion RTs

S-References

Benjamini, Y., Hochberg, Y., 1995. Controlling the False Discovery Rate: A practical and powerful approach to multiple testing. Journal of the Royal Statistical Society. Series B (Methodological) 57, 289–300.

Cheverud, J.M., 2001. A simple correction for multiple comparisons in interval mapping genome scans. Heredity 87, 52–58. doi:10.1046/j.1365-2540.2001.00901.x

Li, J., Ji, L., 2005. Adjusting multiple testing in multilocus analyses using the eigenvalues of a correlation matrix. Heredity 95, 221–227. doi:10.1038/sj.hdy.6800717
